# A Minimal Information Model for Potential Drug-Drug Interactions

**DOI:** 10.3389/fphar.2020.608068

**Published:** 2021-03-08

**Authors:** Harry Hochheiser, Xia Jing, Elizabeth A. Garcia, Serkan Ayvaz, Ratnesh Sahay, Michel Dumontier, Juan M. Banda, Oya Beyan, Mathias Brochhausen, Evan Draper, Sam Habiel, Oktie Hassanzadeh, Maria Herrero-Zazo, Brian Hocum, John Horn, Brian LeBaron, Daniel C. Malone, Øystein Nytrø, Thomas Reese, Katrina Romagnoli, Jodi Schneider, Louisa (Yu) Zhang, Richard D. Boyce

**Affiliations:** ^1^ Department of Biomedical Informatics, University of Pittsburgh, Pittsburgh, PA, United States; ^2^ Intelligent Systems Program, University of Pittsburgh, Pittsburgh, PA, United States; ^3^ Department of Public Health Sciences, Clemson University, Clemson, SC, United States; ^4^ Pharmacy Consulting International (PCI), New York City, NY, United States; ^5^ Department of Software Engineering, Bahçeşehir University, Istanbul, Turkey; ^6^ Clinical Data Science, AstraZeneca, Cambridge, United Kingdom; ^7^ Institute of Data Science, Maastricht University, Maastricht, Netherlands; ^8^ Department of Computer Science, Georgia State University, Atlanta, GA, United States; ^9^ Fraunhofer Institute for Applied Information Technology, RWTH Aachen University, Aachen, Germany; ^10^ Department of Health Outcomes and Biomedical Informatics, University of Florida, Gainesville, FL, United States; ^11^ Mayo Clinic, Rochester, MN, United States; ^12^ Open Source Electronic Health Record Alliance, Washington, DC, United States; ^13^ IBM Research, Yorktown Heights, NY, United States; ^14^ The European Bioinformatics Institute, Birney Research Group, London, United Kingdom; ^15^ Genelex Corporation, Seattle, WA, United States; ^16^ School of Pharmacy, University of Washington, Seattle, WA, United States; ^17^ Southeast Louisiana Veterans Health Care System, New Orleans, LA, United States; ^18^ Department of Pharmacotherapy, University of Utah, Salt Lake City, UT, United States; ^19^ Department of Computer Science, Norwegian University of Science and Technology, Trondheim, Norway; ^20^ Department of Biomedical Informatics, University of Utah, Salt Lake City, UT, United States; ^21^ School of Information Science, University of Illinois, Champaign, IL, United States

**Keywords:** drug-drug interaction, adverse drug events, minimal information model, clinical informatics, knowledge representation

## Abstract

Despite the significant health impacts of adverse events associated with drug-drug interactions, no standard models exist for managing and sharing evidence describing potential interactions between medications. Minimal information models have been used in other communities to establish community consensus around simple models capable of communicating useful information. This paper reports on a new minimal information model for describing potential drug-drug interactions. A task force of the Semantic Web in Health Care and Life Sciences Community Group of the World-Wide Web consortium engaged informaticians and drug-drug interaction experts in in-depth examination of recent literature and specific potential interactions. A consensus set of information items was identified, along with example descriptions of selected potential drug-drug interactions (PDDIs). User profiles and use cases were developed to demonstrate the applicability of the model. Ten core information items were identified: drugs involved, clinical consequences, seriousness, operational classification statement, recommended action, mechanism of interaction, contextual information/modifying factors, evidence about a suspected drug-drug interaction, frequency of exposure, and frequency of harm to exposed persons. Eight best practice recommendations suggest how PDDI knowledge artifact creators can best use the 10 information items when synthesizing drug interaction evidence into artifacts intended to aid clinicians. This model has been included in a proposed implementation guide developed by the HL7 Clinical Decision Support Workgroup and in PDDIs published in the CDS Connect repository. The complete description of the model can be found at https://w3id.org/hclscg/pddi.

## 1 Introduction

Ensuring that medication therapy occurs safely and to the maximum benefit of any given patient is of great interest to clinicians. One possible threat to patient safety comes from exposure to drug combinations that could interact to cause treatment failure, toxicity, or other unanticipated adverse drug reactions. While some potentially interacting combinations can benefit patients (e.g., by reducing the dose required for an expensive drug), drug interactions are more often a patient safety concern. Clinically important events attributable to drug interaction exposure occur in 5.3–14.3% of inpatients, and are responsible for up to 231,000 emergency department visits each year in the United States alone (Magro et al., 2012; Centers forDisease Control, 2018). A meta-analysis of 13 studies conducted on 3 continents found that 22.2% of hospital admissions associated with an adverse drug event were attributable to drug-drug interactions (interquartile range 16.6–36.0%) (Dechanont et al., 2014).

The development and delivery of clear and concise information about potential adverse events associated with drug-drug interactions is a long-standing clinical informatics challenge. Drug experts generate summaries of potential drug-drug interactions (PDDI) evidence from primary sources including the peer-reviewed scientific literature, drug product labeling, and case reports. These knowledge artifacts reach clinicians in the form of published drug information compendium, clinical decision support rules, and interaction checking applications. However, the knowledge content of the numerous resources describing PDDIs are far from uniform, with variations in goals and practice (Romagnoli et al., 2017) leading to significant discrepancies between resources (Wang et al., 2010; Saverno et al., 2011; Ayvaz et al., 2015; Ekstein et al., 2015; Fung et al., 2017). For example, a recent study comparing potential psychiatric drug interactions from six drug interaction database programs found agreement on the PDDI category for only 66% of 100 drug pairs. Fung et al compared 8.6 million unique pairs across 3 commercial PDDI knowledge bases and found that only 5% of PDDIs were mentioned in all 3KBs (Fung et al., 2017). We think a major issue underlying these discrepancies is that there is currently no broadly accepted standards to guide knowledge artifact creators in the organization and content of PDDI information. Given the costs of adverse events associated with drug interactions (Magro et al., 2012; Dechanont et al., 2014; Centers for Disease Control, 2018), improvements in the quality and utility of available information should contribute to improved care and reduced costs. As a means of bridging this gap, we have developed a minimal information model capable of presenting a common schema for describing evidence surrounding a PDDI.

Providing clinical guidance on the management of potential drug-drug interaction evidence requires synthesis and interpretation of multiple, possibly conflicting claims from evidence sources providing various levels of rigor and detail, including new drug announcements, published results of controlled trials, and spontaneous adverse event reports. Our investigation of the practices of experts involved in evaluating drug-drug interaction evidence found a range of personalized practices for both interpretation and synthesis of recommendations (Romagnoli et al., 2017). These variations in process and interpretation of evidence hinder sharing and comparison of recommendations, perhaps contributing to the aforementioned lack of consistency across information resources. Despite these variations, previous efforts established some recommended best practices for describing PDDIs. In 2005, van Roon, et al. (van Roon et al., 2005) published a model including a five-stage rating of evidence quality and a six-stage rating of seriousness of adverse events. A more recent effort by Seden et al. adapts the widely used GRADE (Grading of Recommendations Assessment, Development and Evaluation) methodology to evaluate PDDIs (Seden et al., 2017). This method is used by the various disease focused PDDI knowledge resources created by a collaborative effort led by the University of Liverpool (University of Liverpool, 2020a; University of Liverpool, 2020b; University of Liverpool, 2020c; University of Liverpool, 2020d). The Drug Interaction Probability Scale (DIPS) (Horn et al., 2007) provides a widely adopted structured means of assessing the likelihood of a drug-drug interaction in a specific case.

A series of efforts have attempted to build upon these tools to develop consensus models for PDDI information. A 2011 Delphi study involving 69 experts identified five key descriptors (patient status, probability of occurrence, risk factors, reaction severity, and evidence quality) (Riedmann et al., 2011). A conference series conducted in 2013–2014 developed usability recommendations for drug-drug interaction clinical decision support (Payne et al., 2015), evidence evaluation recommendations (Scheife et al., 2015), and guidance for selecting PDDIs for inclusion in clinical decision support systems (Tilson et al., 2016). A 2016 review of PDDI conceptual models identified 15 approaches with wide variations in granularity and coverage of key elements (Herrero-Zazo et al., 2016).

Building on all of these efforts, our goal was to develop community consensus around a common core set of elements to be included in PDDI knowledge artifacts. The new minimal information model would be used by drug experts to ensure that the PDDI knowledge artifacts they create have the core relevant information needed by the clinician end users. As a minimum information model, it should provide sufficient structure to be broadly useful without requiring substantial curatorial effort. Also, the minimal information model should focus exclusively on what information is to be collected, without addressing how it should be formatted or stored. This approach promotes flexibility and ease of use at the expense of possibilities for computational analysis (Brazma, 2009).

Critical to the domain of PDDIs, the information model should also help creators of PDDI knowledge artifacts include contextual factors that can help clinicians who use the artifacts to more specifically apply the knowledge to specific patient cases. This is because the specificity of a PDDI knowledge artifact to individual patient characteristics play a major role in acceptance of its recommendations (van der Sijs et al., 2006; Seidling et al., 2014). Using examples from PDDI clinical decision support, an *in situ* qualitative study of prescribers’ interaction with electronic medication alerts showed that prescribers bypassed the alert and then searched for patient-specific data that they needed when alerts failed to provide contextual information (Russ et al., 2012). Similarly, quality improvement projects have found that making PDDI alerts more appropriate to clinical context can improve alert acceptance (Daniels et al., 2019).

## 2 Methods

Development of the PDDI minimal information model was conducted under the auspices of the Health Care and Life Sciences Community Group (HCLSCG) of the World Wide Web Consortium (W3C - acronyms used this paper are summarized in Table 1). As a volunteer group, HCLSCG brings together experts interested in the use of Semantic Web tools in both medical and research applications. (Health Care and Life Sciences Community Group, 2018). Working with members of the HCLSCG group, including participants with both technical and clinical experience, we took a user-centered design approach to development of the minimal information model through four complementary activities: the selection of PDDIs used to inform the design of the minimal information model; the identification of user stories to define requirements; the construction of definitions for the elements of the information model; and the development of preliminary knowledge representation guidelines for the minimal information model.

**TABLE 1:** Acronyms, abbreviations, and technical terms used in this paper.

Term	Definition
DDI	Drug-drug interaction - usually referring to events that have occurred in patients
PDDI	Potential drug-drug interaction
HL7	Health level 7 international, an electronic health information standards non-profit
CDS	Clinical decision support - predictive models, information tools, and other electronic health records components designed to help clinicians choose the best course of action while providing patient care

The team started working collaboratively on the minimum information model in January of 2016. Discussion occurred using monthly “all-team” web meetings and additional web meetings focused on the development of use cases and exemplar PDDIs. After agreeing on the primary goals of the project, the team began a detailed discussion of information items mentioned in published consensus recommendations (Payne et al., 2015; Scheife et al., 2015) and development of real-world use cases. The team then used anonymous online surveys followed by team discussions to arrive at consensus on which information items to include in the minimum information model and the formal and user-centered definitions for the information items. A public draft version of the Community Group report was continuously edited as suggestions and disagreements were discussed and resolved. As a report reflecting consensus among the team reached maturity, drafts were sent out to the greater W3C community listserv for comments until a two week period passed without further suggestions. The final report was published in May of 2019 and is available online (https://w3id.org/hclscg/pddi). The next subsections summarize the methods for developing key knowledge artifacts included in the report. Further details on the information model’s development are available in the Appendix of the online report.

### 2.1 Decision Trees

Prior work by participants in the interest group sought to develop evidence-based clinical algorithms that consider a patient’s electronic health record information to provide a clinician with actionable information tailored to the patient’s specific context. Nelson et al. (2017); Rosko et al. (2017); The algorithms are formulated as decision trees, each containing a series of binary questions designed to explicitly constrain the decisions needed to interpret the appropriate response to a PDDI, leading to any of three different recommended actions—no special precautions; assess risk and take action if necessary; and use only if benefits outweigh the risk. Each decision tree should therefore result in a complete and unambiguous process for describing a PDDI.

The team developed decision trees for 14 PDDIs identified through an iterative process involving regular meetings with focused sub-teams. The 14 PDDIs (Table 2) were selected to demonstrate how an information model might help address known challenges with PDDI evidence. For example, two interactions (tamoxifen—paroxetine and potassium - potassium-sparing diuretics) were chosen to represent situations where information about the interaction can (and should) be contextualized for specific patients or clinical circumstances. The interaction between monoamine oxidase inhibitors - and indirect sympathomimetics was chosen because, the it applies for all drugs belonging to both drug classes. In contrast, tyrosine kinase inhibitors—proton pump inhibitors was chosen because the interaction is only relevant for kinase inhibitors that have pH dependent absorption.

**TABLE 2:** A total of 14 PDDI decision trees were created to represent different situations that developers of PDDI knowledge artifacts face. Knowledge artifacts following the minimum information model were created for each PDDI using logic flow diagrams, narrative explanations, and citations. Ten knowledge artifacts have been deposited in Zenodo. The other four are available from the authors upon request.

Exemplar potential drug-drug interactions	Drug or drug class 1	Drug or drug class 2	Doi
Can (and should) be contextualized for specific patients or clinical circumstances	Tamoxifen	Paroxetine	10.5281/zenodo.1471714
	Potassium (KCL)	Potassium-sparing diuretics	
Applies at the class level	Monoamine oxidase inhibitors (MAOIs)	Indirect sympathomimetics	10.5281/zenodo.1472473
Does not apply at the class level	Tyrosine kinase inhibitors	Proton pump inhibitors	10.5281/zenodo.1472469
The mechanism is known and is pharmacokinetic	Warfarin	CYP2C9 inhibitors (ie. Bactrim)	10.5281/zenodo.1472464
	Digoxin	Cyclosporin	10.5281/zenodo.4327204
The mechanism is known and is pharmacodynamic	Epinephrine	Beta-blockers	10.5281/zenodo.1472485
The mechanism is not well elucidated/known	Warfarin	Ifosfamide/Etoposide	10.5281/zenodo.1472471
The evidence supporting the interaction is strong	Epinephrine	Beta-blockers	10.5281/zenodo.1472485
	Simvastatin, atorvastatin, lovastatin	Clarithromycin	10.5281/zenodo.1472460
The evidence supporting the interaction is weak	Warfarin	Antibiotics for which it is uncertain if inhibition of CYP2C9 affects warfarin	10.5281/zenodo.1472471
The frequency of exposure data is available	Warfarin	Non-steroidal anti-inflammatory drugs (NSAIDs)	10.5281/zenodo.1472475
The frequency of exposure data is not available	Simvastatin	Fluconazole	10.5281/zenodo.1472441
The frequency of adverse event data is available	Spironolactone	Potassium supplements	Merged into 10.5281/zenodo.4327267
The frequency of adverse event data is not available	Simvastatin	Fluconazole	10.5281/zenodo.1472441
The recommended action is “monitor” or “take note”	Potassium (KCL)	Potassium-sparing diuretics	10.5281/zenodo.4327267
The recommended action is “avoid”	Monoamine oxidase inhibitors (MAOIs)	Indirect sympathomimetics	10.5281/zenodo.1472473
The recommended action is a clear alternative drug and dose	Simvastatin	Amiodarone	10.5281/zenodo.1472434

Analyses of questions needed to build the 14 decision trees, and their answers, informed selection of items for inclusion in the minimal information model. As part of this process, value sets for categories of medications (aldosterone antagonists, non-steroidal anti-inflammatory drugs, etc.) and clinical conditions (gastrointestinal bleeding, intracranial hermorrhage, etc.) were developed for several of the PDDI decision trees. Value sets are collections of codes from one or more terminologies that are useful for representing the specific drugs, conditions, observations, or other items of interest that have been coded in electronic health records. For example, a value set for paroxetine could include all clinical drug codes from the RxNorm terminology that are used when the electronic health records stores a prescription for any form of that medication. Value sets can be extensional, meaning that all included codes are simply listed, or intensional, meaning that the codes are specified using a workflow or logical definition. The value sets we created for the note were extensional and are available in the appendix of https://w3id.org/hclscg/pddi and also at the National Library of Medicine’s Value Set Authority Center (https://vsac.nlm.nih.gov/—search for value sets created by the “Meaningful Drug Interaction Alerts” organization).

### 2.2 User Stories

As our goal was to develop an information model to help PDDI knowledge artifact developers provide the information necessary to help clinicians make prescribing choices informed by accurate information regarding PDDIs, we developed a set of user stories to demonstrate the application of the minimal information model and potential utility for clinical challenges. These user stories focused on the challenges facing individuals involved in collating drug-drug interaction information, developing treatment plans, building clinical decision support systems, and otherwise providing appropriate information to clinicians. Each user story discussed instances where PDDI evidence described in the minimal information model might have been necessary to help provide effective care. Thus, pharmacists, physicians, nurses, librarians, and systems developers were considered to be potential users of the PDDI information model.

Initial stakeholder descriptions and scenarios were developed and used as the basis for further brainstorming with the assistance of a user experience expert. These efforts led to the development of a master list of tasks, users, information needs, and barriers to drug-drug interaction based decision-making in a variety of situations. A core set of user types was selected for development of user stories based on the scope of the minimum information model.

User stories for these core user types were developed based on an initial information needs list created by interest group members. This list was supplemented based on the results of a recent study of PDDI information needs of drug information compendium editors (Romagnoli et al., 2017). Where possible, user stories were based on PDDIs suggested by the interest group’s PDDI experts. All user stories were reviewed during team meetings to solicit feedback and comments. Based on interest group member suggestions, user stories were edited for clinical relevance, accuracy, and appropriateness. Elements of user stories were highlighted based on a color-coded key to indicate the minimum information model information item in question. Medication reconciliation use cases were specifically targeted as a compelling demonstration of the importance of the elements of the minimal information model.

### 2.3 Definitions

After agreeing on a set of conceptual information items for the information model that were grounded in the user stories, we developed a draft definitions for them. The definitions were informed by entities present in the Drug-Drug Interaction Evidence Ontology (DIDEO) (Brochhausen et al., 2014) and the Drug-Drug Interactions Ontology (DINTO) (Herrero-Zazo et al., 2015). These draft definitions were then shared with all team members who were asked to provide suggestions for clarification. The final definitions, agreed upon by all report authors, were added to a new ontology called MPIO (http://www.ontobee.org/ontology/MPIO) for use in the minimal information model.

## 3 Results

The complete model, including detailed use cases, two examples of application to PDDI (narrative and JSON), and other supporting information, can be found at https://w3id.org/hclscg/pddi.

### 3.1 User Stories

Twelve user stories involving eight types of users focused on key clinical challenges illustrate the potential uses of the minimal information model (Table 3). Three of these user stories focus on the critical task of medication reconciliation; others covered a range of topics, including treatment planning, evaluation of management options, screening for drug-drug interactions, and synthesis for dissemination. Several classes of user stories are repeated from the perspectives of different stakeholders, including physicians, nurses, pharmacists, drug compendium editors, librarians, and clinical decision support system staff. All user stories are annotated to indicate items from the minimal information model (an example is given in Table 4). As the initial focus was on clinical needs, researchers and regulators are not included explicitly in these initial user stories.

**TABLE 3:** Goals and users for user stories illustrating the use of the minimal information model. The complete user stories are at https://w3id.org/hclscg/pddi.

Goal	User(s)	# Of stories
Treatment planning	Physician	3
Evaluation of management options	Physician, pharmacist	2
Screening for PDDIs	Nurse	1
Synthesizing PDDI evidence	Drug compendium editor, health science librarian	2
Translating evidence to a clinical decision support tool	Systems analyst, content specialist	1
Medication reconciliation	Hospital & consulting pharmacist, physician	3

**TABLE 4:** Treatment planning user story example, annotated with instances of information model items.

Kathleen is a physician who is treating a patient who has a ventricular arrhythmia. Kathleen would normally prescribe amiodarone for this particular patient, but he is being treated with simvastatin for dyslipidemia, and she knows that a *potentially serious interaction*[Seriousness] may occur leading to *rhabdomyolysis*[Clinical Consequence]. Kathleen wants to know *what the patient's risk factors are for rhabdomyolysis*[Contextual evidence/modifying factors], what the benefits and risks would be to *switching him to an alternative statin*[Recommended Actions], and if amiodarone is not the best option for this patient, *what alternatives to amiodarone*[Recommended Actions] exist for this patient, and what the available *evidence*[Evidence] shows in terms of ventricular arrhythmia patient outcomes.

### 3.2 Elements of the PDDI Minimal Information Model

The minimal information model contains 10 core elements (drugs involved, clinical consequences, seriousness, operational classification statement, recommended action, mechanism of interaction, contextual information/modifying factors, frequency of exposure to the PDDI, frequency of harm for persons who have been exposed to the PDDI, and evidence about a suspected drug-drug interaction). The definitions and eight detailed best practice recommendations are provided in https://w3id.org/hclscg/pddi. In this paper, the definitions are presented using two example PDDIs (warfarin and non-steroidal anti-inflammatory drugs and tyrosine-kinase inhibitors and proton pump inhibitors) in Tables 5, 6. A brief description of each core element provided here.

**TABLE 5:** The minimal information model and example elements as applied to potential interactions between Warfarin and non-steroidal anti-inflammatory drugs (NSAIDS).

Element	Required?	Description	Example
Drugs involved	✓	At least two participating drug products, classes, or active ingredients	Warfarin and non-steroidal anti-inflammatory drugs (value sets at https://w3id.org/hclscg/pddi)
Clinical consequences	✓	Possible health outcomes, described as specifically as possible, ideally from a terminology such as ICD10 or SNOMED-CT, or from a public value set	Increased risk of bleeding, including gastrointestinal bleeding, intercranial hemorrhage, and cerebral hermorrhage (value sets at https://w3id.org/hclscg/pddi)
Seriousness	✓	A PDDI consequence is serious if it may result in death or other potentially life-threatening or impairing situations	Bleeding is serious because it may result in death, life-threatening hospitalization, and disability
Operational classification statement	✓	Specific management strategies for a given patient context, including possibilities such as avoiding the combination of drugs, use only under special circumstances, minimize risk, monitor, no special precautions, and ignore Hansten et al. (2001)	Consider alternatives: Alternatives may be available that are less likely to interact
Recommended action		Evidence-based approaches to minimize risks, including alternate drugs	If the NSAID is being used as an analgesic or antipyretic, consider alternatives such as acetaminophen and monitor INR if use continues. For severe pain, consider opioids in place of the NSAID
Mechanism of interaction	✓	The process(es) involved in the interactions between the drugs in question	Anti-platelet effects of NSAIDS can increase bleeding risks when combined with anticoagulants such as warfarin
Contextual information/modifying factors	✓	Demographic, clinical, drug-delivery details (route, product), or other details that might impact the risk of consequences of a DDI	NSAID is topical diclofenac; co-administration of proton pump inhibitors or misoprostol; history of ulcers or upper gastrointestinal bleeding (value sets at https://w3id.org/hclscg/pddi)
Evidence	✓	Information supporting or refuting the existence of the interaction in humans. Potential evidence sources include clinical study data, observations, physiological experiments, or mechanistic predictions	Corticosteroids and aldosterone antagonists have been shown to increase the risk of upper gastrointestinal bleeding in patients on NSAIDS Masclee et al. (2014) (value sets at https://w3id.org/hclscg/pddi)
Frequency of exposure to the PDDI		The proportion of individuals within a cohort who have been administered the drugs involved, expressed over a given time period	Concomitant NSAIDs occur with 24.3% of warfarin courses of therapy. Malone et al. (2005)
Frequency of harm for persons who have been exposed to the PDDI		The fraction of individuals exposed to the drug combination who experience harm	The relative risk of upper GI bleeding with concurrent warfarin and NSAID use is 2.9–3.3 higher than compared to a patient who takes warfarin alone. Chung et al. (2005)

**TABLE 6:** The minimal information model and example elements as applied to potential interactions between Tyrosine kinase inhibitors and Proton Pump Inhibitors.

Element	Required?	Description	Example
Drugs involved	✓	At least two participating drug products, classes, or active ingredients	Tyrosine kinase inhibitors and proton pump inhibitors (value sets at https://w3id.org/hclscg/pddi)
Clinical consequences	✓	Possible health outcomes, described as specifically as possible, ideally from a terminology such as ICD10 or SNOMED-CT, or from a public value set	Decreased efficacy relative to treatment for chronic myeloid leukemia (value sets at https://w3id.org/hclscg/pddi)
Seriousness	✓	A PDDI consequence is serious if it may result in death or other potentially life-threatening or impairing situations	A decrease in chronic myeloid leukemia treatment efficacy is a serious potential clinical consequence because it can result in death, life-threatening hospitalization, and disability.
Operational classification statement	✓	Specific management strategies for a given patient context, including possibilities such as avoiding the combination of drugs, use only under special circumstances, minimize risk, monitor, no special precautions, and ignore Hansten et al. (2001)	If the TKI is imatinib or ponatinib - No special precautions; if the TKI is nilotinib - assess risk and take action if necessary; if the TKI is bosutinib or dasatinib—use only if benefit outweighs risk.
Recommended action		Evidence-based approaches to minimize risks, including alternate drugs	Antacids and H2 antagonists may be considered if TKI is given 2 h before the antacid/H2 antagonist
Mechanism of interaction	✓	The process (es) involved in the interactions between the drugs in question	The TKIs demonstrate pH dependent absorption for oral administration which may result in decreased efficacy when given concomitantly with medications that increase gastric pH.
Contextual information/modifying factors	✓	Demographic, clinical, drug-delivery details (route, product), or other details that might impact the risk of consequences of a DDI	Imatinib and ponatinib AUCs are not appreciably decreased by PPI co-administration Millennium Pharmaceuticals, Inc. (2020); Egorin et al. (2009). For nilotinib, esomeprazole (a PPI) decreased the nilotinib AUC by 34% but a retrospective study has shown no difference in cytogenetic response rates for patients taking PPIs. Yin et al. (2012); Novartis (2020) product labeling recommends avoiding use of concomitant PPIs with bosutinib or dasatinib. Pfizer (2020); E.R. Squibb and Sons, L.L.C. (2018)
Evidence	✓	Information supporting or refuting the existence of the interaction in humans. Potential evidence sources include clinical study data, observations, physiological experiments, or mechanistic predictions	See the evidence citations above.
Frequency of exposure to the PDDI		The proportion of individuals within a cohort who have been administered the drugs involved, expressed over a given time period	Unknown
Frequency of harm for persons who have been exposed to the PDDI		The fraction of individuals exposed to the drug combination who experience harm	Unknown

#### 3.2.1 Drugs Involved

Minimal information model PDDI Knowledge artifacts need to indicate the drugs involved in the PDDI with either extensional or intensional value sets consisting of concepts from widely adopted vocabularies such as RxNorm (National Institutes of Health, 2020a), or the Anatomical Therapeutic Chemical Classification System (WHO Collaborating Center for Drug Statistics Methodology, 2020) for drugs.

#### 3.2.2 Clinical Consequences

Minimal information model PDDI Knowledge artifacts need to indicate the known or anticipated clinical consequences of exposure to the PDDI. This is important because many knowledge sources, including drug product labeling, report PDDIs that are based on pharmacologic properties (e.g., pharmacokinetics) without specifying what a potential clinical consequence. Without a clear clinical consequence, it is very hard for clinicians to asses the potential risks to the patient. Where possible, knowledge artifacts should indicate the clinical consequences using either extensional or intensional value sets consisting of concepts from widely adopted vocabularies such as ICD-10 (World Health Organization, 2020) or SNOMED-CT (SNOMED International, 2020).

#### 3.2.3 Seriousness

Minimal information model PDDI Knowledge artifacts need to report if each known or anticipated clinical consequence is serious or not. Serious clinical consequences include death, life-threatening hospitalization, congenital anomaly, disability, or permanent impairment or damage. The seriousness entity was added to the model to bridge the gap between the community of pharmacy practitioners and creators of drug compendia and drug-drug interaction software tools, who commonly discuss PDDIs in terms of severity (Abarca et al., 2004; Roblek et al., 2015; Romagnoli et al., 2017), and the pharmacovigilance community, who describe events leading to death, hospitalizaiton, or persistent harm as being serious (WHO-Uppsala Monitoring Centre, 2020). The concept of severity is not included in the model. In place of severity, the report recommends the use of an operational classification statement (described below) which is thought be generally more clear and useful in a clinical decision making context.

An Operational Classification Statement is short risk classification statement that suggests a specific management criteria for a specific patient context. Minimal information model PDDI Knowledge artifacts need to report at least one such statement. The report suggests the use of the following categories from the OpeRational ClassificAtion of Drug Interactions system (Hansten et al., 2001):

• Avoid Combination (Risk of combination outweighs benefit)
• Usually Avoid Combination (use only under special circumstances)
• Interactions for which there are clearly preferable alternatives for one or both drugs.
• Interactions to avoid by using an alternative drug or other therapy unless the benefit is judged to outweigh the increased risk.
• Minimize Risk (Assess risk and take one or more of the following actions if needed)
• Consider alternatives: Alternatives may be available that are less likely to interact.
• Circumvent: Take action to minimize the interaction (without avoiding combination).
• Monitor: Early detection can minimize the risk of an adverse outcome.
• No Special Precautions (Risk of adverse outcome appears small)
• Ignore (Evidence suggests that the drugs do not interact)

Adopters of the minimum information model can expand on these or use their own operational classification statement system. In either case, recommended actions should be linked, if possible, to justifying evidence.

The Recommended Action element can be used independently, as a suggestion for dealing with any instance of the PDDI, or as modified by some contextual information/modifying factors (see below), to indicate guidance specific to patient factors or given drug formulations. A well written recommended action provides an evidence-based strategy to mitigate the potential clinical consequences of a drug-drug interaction such as a drug change, adjust drug dose, and monitor lab values. It should provide specific detailed suggestions that complement the Operational Classification Statement. The three basic recommended actions can be the same as the operational classification statements (No special precautions, Assess risk and take action if necessary, and Use only if benefit outweighs risk). Adopters of the minimum information model could expand on these or use their own system. In either case, recommended actions should be linked, if possible, to justifying evidence.

#### 3.2.4 Mechanism

If known, a minimum information model PDDI knowledge artifact needs to include a statement about the mechanism of the PDDI. The description should be written for a clinician audience and include details that help the clinician decide what course of management action to take. To reduce ambiguity, the description may refer to specific drugs or health conditions using codes or value sets from widely used terminologies.

Contextual information/modifying factors are necessary for alerts that are both sensitive and specific. Like clinical consequences, minimum information model PDDI knowledge artifacts should indicate each known factor be stated as specifically as possible.

Many potential interactions are based on a small number of case reports or inference from pharmacologic properties. The two information items Frequency of exposure to the PDDI and Frequency of harm for persons who have been exposed to the PDDI help clarify if any evidence exists on the public health impact of the PDDI. While potentially very helpful for making a risk/benefit assessment about exposure to a PDDI, accurate frequency counts for may be unavailable in most cases. If so, “unknown” should be indicated to convey the limits of available information. In this way, minimum information model knowledge artifacts can help make apparent evidence gaps in need of further clinical research.

#### 3.2.5 Evidence About a Suspected Drug-Drug Interaction

A minimum information model PDDI knowledge artifact needs to include evidence support for or in refutation of a given drug-drug interaction in humans. This evidence could potentially include data resulting from clinical studies, clinical observation, physiological experiments, or extrapolation based on drug-drug interaction mechanisms. The report’s best practice recommendations indicate that links to evidence are particularly essential to the artifact’s operational classification statement(s) recommended action(s).

A more detailed description of the model and its application can be found in the W3C note, available at https://w3id.org/hclscg/pddi.

### 3.3 A Word About Knowledge Representation

This section is intended for readers interested in knowledge representation aspects of the information model and could be skipped by non-interested readers. Ideally, representations of PDDI information would be fully-computable, expressed in a formal representation with well-specified semantics. However, the ambiguity of existing free-text descriptions of medications, interactions, contextual information, and other key elements makes this a long-term vision rather than a short-term goal. To maximize opportunities for automated analysis, the PDDI information model should be described using existing terminologies and ontologies wherever possible, while acknowledging that accurate capture of free text details can be useful as a last resort. Specific suggestions include:

#### 3.3.1 Use of Biomedical Ontologies to Represent Items from the PDDI Information Model

All of the 10 core elements of the information model are represented in the Minimum PDDI Information Ontology (MPIO) (Boyce et al., 2019). MPIO classes extend classes from the Basic Formal Ontology (BFO) (Arp et al., 2015), supporting integration with the DIDEO (Brochhausen et al., 2014) and DINTO (Herrero-Zazo et al., 2015) ontologies mentioned above, as well as with ontologies relevant to descriptions of experiments, such as the Ontology for Biomedical Investigations (Bandrowski et al., 2016) and adverse events (He et al., 2014).

#### 3.3.2 Representation of Model Elements as Information Content Entities

As statements of possible relationships, PDDI models do not specifically refer to objects that exist in the world. The theoretical nature of these statements is somewhat at odds with the accepted use of the BFO to describe existing objects. To avoid this difficulty, PDDIs are modeled as subclasses of the BFO-compliant class information content entity from the information artifact ontology (IAO) (Ruttenberg, 2020), indicating that each element is a statement that conveys some information, rather than a statement about an existing interaction in the world.

#### 3.3.3 Use of Multiple Terminologies at Multiple Granularities

Drugs, contextual information/modifying factors, mechanisms of interaction, clinical consequences, and other elements of the PDDI model may be represented by multiple concepts from multiple sources, including RxNorm (National Institutes of Health, 2020), ATC (WHO Collaborating Center for Drug Statistics Methodology, 2020), ICD10 (World Health Organization, 2020), SNOMED-CT (SNOMED International, 2020). These concepts might be represented with varying degrees of specificity, ranging from specific products to broad classes of drugs, such as NSAIDs. Although the minimal information model suggests that concepts from accepted vocabularies should be used whenever possible, specific sources and granularities are not specified. Rather, concepts should be presented unambiguously through the combination of identifiers both for the source vocabulary and for the specific concept. If a potential interaction involves multiple drugs or outcomes that cannot be represented by a single concept, value sets might be used to describe multiple concepts. In cases where concepts cannot be represented by ontology terms, textual descriptions might be used as a last resort. When used, free-text summaries should be a specific as possible. Although not ideal, these unstructured descriptions are preferable to the omission of potentially useful information.

Two example representations of PDDI information in the Javascript Object Notation (JSON) format are provided in the note. As a data transfer format often used for communicating information between web clients and server applications, these JSON models illustrate how these PDDI models might be conveyed to web-based clinical applications. These examples also demonstrate how instances of the information model could be used to represent PDDI information. The note does not provide any automated validation or construction tools at this time. Such enhancements will be the focus of future work.

## 4 Discussion

PDDIs present significant challenges in information representation and interpretation. Variations in descriptions of drugs, adverse events, sources of evidence, and patient characteristics are often confounded by challenges associated with incomplete information and evidence from differing sources, leading to differences in interpretation that might leave clinicians without clear, actionable guidance.

The proposed PDDI minimal information model builds on both prior work in minimal information models (Brazma et al., 2001; Taylor et al., 2008; McQuilton et al., 2016) and discussions with drug-drug interaction experts (Payne et al., 2015; Tilson et al., 2016), presenting a set of 10 items identified as most critical for conveying available understanding of PDDIs. These data elements, along with eight best practice recommendations and the proposed guidance on knowledge representation, present a first step toward a broader goal of fully-computable and semantically well-specified representations for drug-drug interaction information.

The PDDI minimal information model is already having an impact on health information standards and technology development activities. The model has been adopted by the HL7 Clinical Decision Support Workgroup, which works within the HL7 organization to develop clinical decision support standards (HL7, 2020a). The PDDI model has also be included in a new PDDI clinical decision support implementation guide that the HL7 community recently successfully balloted as a reference standard for trial use (Nguyen et al., 2019; HL7 Clinical Decision Support Workgroup, 2020). The implementation guide provides guidance for actionable PDDI alerts using sharable clinical decision support (CDS) artifacts that adhere to the minimum information model and the health information technology standards CDS Hooks (HL7, 2020b), Clinical Quality Language (CQL) (HL7, 2020c), and Fast Healthcare Interoperability Resources (FHIR). (Bender and Sartipi, 2013; Mandel et al., 2016; HL7, 2020b; HL7, 2020c; HL7, 2020d). Together, these efforts provide technical guidance for representing clinical data as stored in electronic medical records, querying systems for relevant data, and building tools that might integrate pertinent information into clinical workflows. Adoption of the PDDI model by these efforts provides a pathway for the inclusion of the PDDI information in widely-used clinical tools.

Realizing the vision of meaningful and highly actionable PDDI information will require additional work aimed at addressing several issues that have been raised by reviewers of early versions of the proposed model. Additional guidance on the distinction between the severity and seriousness elements may be needed to help unfamiliar users populate and interpret those terms appropriately. Additional examples, potentially including PDDIs described as being severe but not serious, might be particularly useful in this regard.

Experience with exploration of the warfarin-NSAID PDDI illustrates the importance of providing support for the use of customized value sets. As textual descriptions of PDDIs routinely lump concepts into categories (such as NSAIDs) that are not well-represented in existing ontologies, value sets provide a key tool for enabling appropriate specificity. Although the use of existing value sets developed through community efforts (National Institutes of Health, 2020b) might be the preferred approach, we anticipate that some PDDI descriptions will require novel value sets. A robust PDDI information ecosystem would enable sharing and reuse of such value sets wherever possible.

Although initially focused on meeting clinical needs, the proposed minimal information model might also be used to support broader technical, regulatory, and research goals. The W3C note (https://w3id.org/hclscg/pddi) provides justification for potential uses of the PDDI model for enhancing drug-drug interaction statements in structured product labels. The minimum information model can also be used to point out limitations in available information. Although clearly useful when available, the frequency of harm and frequency of exposure elements will often necessarily be specified as “unknown”. As large-scale observational efforts such as the Observational Health Data Sciences and Informatics (OHDSI) Hripcsak et al. (2015) effort continue to grow, retrospective extraction of such prevalence information from large cohorts is more of a practical possibility. For PDDIs identified through clinical trials, prevalence of harm within participating cohorts might provide information appropriate for these elements. The definition of well-specified models for expressing harm ratios will be an important step toward computable PDDI representations. Future enhancements might include expansion to include additional recommended data items designed to support researchers, regulators, and other perspectives.

Prior work on knowledge representation for PDDI evidence might be used to provide additional structure for the evidence item in the information model. As knowledge schemas designed to represent assertions from the research literature, Micro- and nano-publications (Schneider et al., 2015), might be used to model specific evidence statements. Particularly when combined with semantic classes from the Drug-Drug interaction and drug-drug interaction evidence ontology (Brochhausen et al., 2014) and the Drug-Drug Interactions Ontology (DINTO) (Herrero-Zazo et al., 2015), might bring needed structure appropriate for the interpretation and classification of evidence types and sources.

Appropriate tooling may be necessary to encourage potential users to adopt the PDDI information model. The MIAME model was eventually supported by a range of tools, including an XML markup language (Spellman et al., 2002), a spreadsheet format (Rayner et al., 2006), and the MAGE-TAB collection of tools supporting validation, visualization, and other key tasks (Rayner et al., 2009). The definition of the minimal information model contains preliminary examples of possible XML representations, but more work would be needed to develop a full schema. Similarly, further design work will be needed to support spreadsheet formats, a formal scheme, validation tools, and other support necessary to minimize the burden of completing compliant representations of PDDI information. Such efforts should leverage recent work in semantic metadata annotation (Egyedi et al., 2017).

Shared models of information describing the risks of PDDIs have the potential to improve the applicability of this information to clinical care, thus reducing adverse events, increasing medication safety, and improving outcomes. We present a proposed model containing 10 information items identified through a community consensus process: the drugs involved in a potential DDI, the clinical consequences, seriousness, an operational classification statement, recommended action, mechanism of interaction, contextual information/modifying factors, evidence about the suspected drug-drug interaction, frequency of exposure, and frequency of harm. These items, together with preliminary knowledge representation recommendations, present a first step toward improved collection, curation, and communication of PDDI information. We are committed to working with the community to promote and expand this model, with the goal of developing fully-computable information exchange of PDDI information. Further details are available at https://w3id.org/hclscg/pddi.

## Data Availability

The original contributions presented in the study are included in the article/Supplementary Material, further inquiries can be directed to the corresponding author.
